# Clinical and Imaging Characteristics in Patients with SARS-CoV-2 Infection and Acute Intracranial Hemorrhage

**DOI:** 10.3390/jcm9082543

**Published:** 2020-08-06

**Authors:** Jawed Nawabi, Andrea Morotti, Moritz Wildgruber, Gregoire Boulouis, Hermann Kraehling, Frieder Schlunk, Elif Can, Helge Kniep, Götz Thomalla, Marios Psychogios, Bernd Hamm, Jens Fiehler, Uta Hanning, Peter Sporns

**Affiliations:** 1 Department of Radiology (CCM), Charité-Universitätsmedizin Berlin, Campus Mitte, Humboldt-Universität zu Berlin, Freie Universität Berlin, 14195 Berlin, Germany; elif.can@charite.de (E.C.); b.hamm@charite.de (B.H.); 2 Neurology Unit, ASST Valcamonica, Esine, 25040 Brescia, Italy; andrea.morotti85@gmail.com; 3 Klinik und Poliklinik für Radiologie, Klinikum der Universität (LMU), 81377 Munich, Germany; moritz.Wildgruber@med.uni-muenchen.de; 4 Pediatric Radiology Department, Necker Enfants Malades & GHU Paris, Sainte-Anne Hospital, Institut de Psychiatrie et Neurosciences de Paris (IPNP), UMR S1266, INSERM, Université de Paris, 75015 Paris, France; gregoireboulouis@gmail.com; 5 Department of Radiology, University Hospital Muenster, 48149 Muenster, Germany; Hermann.Kraehling@ukmuenster.de; 6 Department of Neuroradiology, Charité—Universitätsmedizin Berlin, Corporate Member of Freie Universität Berlin, Humboldt-Universität zu Berlin, and Berlin Institute of Health, 14195 Berlin, Germany; frieder.schlunk@charite.de; 7 Department of Diagnostic and Interventional Neuroradiology, University Medical Center Hamburg Eppendorf, 20251 Hamburg, Germany; h.kniep@uke.de (H.K.); fiehler@uke.de (J.F.); u.hanning@uke.de (U.H.); peter.sporns@hotmail.de (P.S.); 8 Department of Neurology, University Medical Center Hamburg-Eppendorf, 20251 Hamburg, Germany; thomalla@uke.de; 9 Department of Neuroradiology, Clinic for Radiology and Nuclear Medicine, University Hospital Basel, 4031 Basel, Switzerland; marios.psychogios@usb.ch

**Keywords:** intracranial hemorrhage, SARS-CoV-2, COVID-19, imaging characteristics

## Abstract

Background and Purpose: Intracranial hemorrhage has been observed in patients with severe acute respiratory syndrome coronavirus 2 (SARS-CoV-2) infection (COVID-19), but the clinical, imaging, and pathophysiological features of intracranial bleeding during COVID-19 infection remain poorly characterized. This study describes clinical and imaging characteristics of patients with COVID-19 infection who presented with intracranial bleeding in a European multicenter cohort. Methods: This is a multicenter retrospective, observational case series including 18 consecutive patients with COVID-19 infection and intracranial hemorrhage. Data were collected from February to May 2020 at five designated European special care centers for COVID-19. The diagnosis of COVID-19 was based on laboratory-confirmed diagnosis of SARS-CoV-2. Intracranial bleeding was diagnosed on computed tomography (CT) of the brain within one month of the date of COVID-19 diagnosis. The clinical, laboratory, radiologic, and pathologic findings, therapy and outcomes in COVID-19 patients presenting with intracranial bleeding were analyzed. Results: Eighteen patients had evidence of acute intracranial bleeding within 11 days (IQR 9–29) of admission. Six patients had parenchymal hemorrhage (33.3%), 11 had subarachnoid hemorrhage (SAH) (61.1%), and one patient had subdural hemorrhage (5.6%). Three patients presented with intraventricular hemorrhage (IVH) (16.7%). Conclusion: This study represents the largest case series of patients with intracranial hemorrhage diagnosed with COVID-19 based on key European countries with geospatial hotspots of SARS-CoV-2. Isolated SAH along the convexity may be a predominant bleeding manifestation and may occur in a late temporal course of severe COVID-19.

## 1. Introduction

The coronavirus disease 2019 (COVID-19) is a worldwide emerging and rapidly evolving situation. As of 1 June 2020, the World Health Organization (WHO) confirmed 6,057,853 cases of COVID-19 with 371,166 deaths globally [1]. The novel severe acute respiratory syndrome coronavirus 2 (SARS-CoV-2) was reported to have symptoms resembling that of severe acute respiratory syndrome CoV (SARS-CoV) in 2003 [2]. Evidence is increasing from clinical studies across China and Europe that an infection with SARS-CoV-2 does not only lead to pneumological complications and systemic inflammatory reactions, but rather also to neurological symptoms [3]. A comprehensive review of the neurological disorders reported during the current COVID-19 pandemic demonstrates that infection with SARS-CoV-2 affects the central nervous system (CNS), the peripheral nervous system (PNS), and the skeletal muscles [3,4]. Moreover, nervous system manifestations were significantly more common in severe infections compared with non-severe infections. Increased D-dimer levels were observed in patients with a severe respiratory history in China. D-dimers increase in sepsis, but can also indicate an activated coagulation system, and thus an increased tendency to thrombosis and coagulopathy [5]. This activation is also described for SARS-CoV-1 and could also promote infarction in CoV-2 as increased D-dimer levels have been observed in previous SARS infections [5]. Evidence of viral infection of the endothelial cell and diffuse endothelial inflammation was reported in a series of patients with COVID-19 [6]. Thus, endothelial inflammation and acquired coagulopathy may increase the risk of ischemic cerebrovascular events. A recently published neuroimaging study from Mahammedi et al. demonstrated that imaging features were dominated by acute ischemic infarcts and intracranial hemorrhages in COVID-19 patients [7]. Especially, ischemic stroke can be the presenting symptom of COVID-19 occurring with a 7.5-fold higher risk in comparison to an influenza virus infection [3,8,9]. The occurrence of intracerebral bleeding has also been reported, but important aspects such as the temporal relationship to COVID-19 infection remain unclear [3,7]. The aim of this study was to describe clinical presentation, timing, and imaging features of patients with COVID-19 infection presenting with acute intracranial bleeding, and thereby raise awareness in clinicians treating COVID-19 patients. 

## 2. Methods

### 2.1. Study Population

This was a multicenter retrospective, observational case series at 5 designated European special care centers for COVID-19 (Charité University Hospital Berlin, Germany; Ludwig-Maximilians-University Hospital of Munich, Germany; University Hospital of Muenster, Germany; Université de Paris, France; and University Hospital Basel, Switzerland) and high-volume tertiary stroke centers. The study included 18 consecutive patients admitted between 16 February 2020 and 19 May 2020 with laboratory-confirmed diagnosis of severe acute respiratory syndrome coronavirus 2 infection and acute intracranial hemorrhage. Patients were diagnosed with COVID-19 according to the WHO interim guidance. Retrospective data collection was based on radiology reports of patients with intracranial hemorrhage. Inclusion criteria were defined as (1) patients with acute intracranial hemorrhage on computed tomography (CT) neuroimaging and additional radiological assessment of the chest (2) who were positive for COVID-19 and (3) suffered from acute neurological symptoms at admission or during hospital stay. Each of the scans had an electronic clinical and, if applicable, pathology report associated with it. Electronic reports were reviewed to extract clinical, laboratory, pathology, and demographic data. In detail, a confirmed case of COVID-19 was defined as a positive result on real-time reverse-transcription polymerase chain reaction (PCR) analysis of throat swab and blood specimens. Radiologic assessments included chest and head CT. Patients were excluded if they had a secondary intracranial hemorrhage from head trauma, hemorrhagic transformation of ischemic infarction, brain tumor, cerebral aneurysm, or vascular malformation. Baseline patient characteristics were retrieved from medical records, including symptom onset, Glasgow Coma Scale (GCS) at admission and modified Rankin Scale (mRS) at last medical evaluation or at discharge. Additionally, vascular risk factors (hypertension, diabetes mellitus), laboratory parameters (lactate dehydrogenase, D-dimer), and invasive procedures such as craniectomy or intraventricular drainage placement as well as due to respiratory failure (ventilation, extracorporeal membrane oxygenation) from patients’ clinical records and follow-up CT were obtained. Any missing or uncertain records were collected and clarified through direct communication with health care clinicians. 

COVID-19 severity was defined as mild, regular, or severe/critical based on the 7th edition of “Novel Coronavirus Pneumonia Diagnosis and Treatment Plan”. Patients were grouped into four categories: mild (minor clinical symptoms and absent lung inflammation on chest *X*-ray), regular (fever and respiratory tract symptoms, with visible lung inflammation on imaging), severe (shortness of breath, RR > 30 breaths/min or sPO2 < 93% at rest), and critical (mechanical ventilation, shock, or combined failure of other organs requiring ICU monitoring) [4,10].

Electronic clinical records were reviewed for neurologic symptoms or signs affecting the central or peripheral nervous systems. Patients were then categorized into two groups: “neurological symptoms first” with neurological manifestations upon initial assessment, and “respiratory symptoms first” who developed neurological symptoms greater than 24 h after hospitalization for COVID-19 [4].

This study was approved by the German COVID-19 Research Board and the ethics committee of Charité Berlin (EA1/035/20). Written informed consent was waived by the institutional review boards and the German COVID-19 Research Board owing to the rapid emergence of the disease and the urgent need to collect data. All study protocols and procedures were conducted in accordance with the Declaration of Helsinki. The data that support the findings of this study are available from the corresponding author upon reasonable request.

### 2.2. Image Acquisitions

The non-contrast CT (NCCT) scans were performed using standard clinical parameters with axial <5 mm section thickness. All images were inspected for quality check and excluded in case of severe motion artifacts. Additional computed tomography angiography (CTA) was performed using a highly iodinated contrast medium and NaCl flush after bolus tracking at the level of the ascending aorta with axial <5 mm section thickness.

### 2.3. Image Analysis

NCCT scans were obtained and stored for further evaluation. The type and location of the intracerebral hemorrhage and presence of intraventricular hemorrhage was assessed and documented. The hemorrhage was classified as parenchymal hemorrhage, subarachnoid hemorrhage (SAH), subdural or epidural hemorrhage. Locations of ICH were classified as deep (basal ganglia and thalamic), lobar, brain stem, and cerebellum. 

### 2.4. Statistics

For baseline data, mean and standard deviations (SD) were used for normally distributed data, and median and range for data that were not normally distributed. Categorical variables were expressed as counts and percentages. Continuous variables were compared by using the Wilcoxon rank sum test. Proportions for categorical variables were compared using the *χ*^2^ test. *p*-Values of less than 0.05 were considered significant. Analyses were performed using the statistical software package SPSS version 25^®^ (IBM Corporation, Armonk, NY, USA). Modified Rankin Scores (mRS) were dichotomized into 0–3 and 4–6 because the difference between these 2 categories was medically relevant. Patients with mRS between 0 and 3 maintain some degree of independence in the activities of daily living, while patients with scores >3 need complete assistance [11].

## 3. Results

### 3.1. Demographic and Clinical Characteristics

A total of 18 hospitalized patients with confirmed SARS-CoV-2 infection and intracranial hemorrhage were included in the analysis. Their demographic and clinical characteristics are shown in Table 1. Median age was 49.5 (IQR: 39.5–62.8) with nine female patients (50.0%). Of these patients, 15 (83.3%) had at least one of the following underlying disorders: hypertension (10, (55.6%)) and diabetes (4, (22.2%)). Additional cardiovascular comorbidities were found in six (33.3%), neurovascular comorbidities in one (5.6%), and oncological comorbidities in four patients (22.2%). Eight patients were under anticoagulation (44.4%) and one patient under antiplatelet treatment (5.6%). Median time difference in days between symptom onset and NCCT imaging was 1.5 (IQR: 0–3) and median time difference between admission and NCCT was 11 days (IQR: 9–29.4). Median GCS was 9 (IQR: 3–7). Laboratory parameters were LDH 483.5 U/L (218.0–738.5 U/L), Creatinine 2.06 mg/dL (IQR 1.5–2.8 mg/dL), C-reactive protein mg/L 239.8 (IQR 145.0 mg/dL–377.1 mg/dL), platelets 218.0 × 10^3^/µL (IQR 108.5–445.3 × 10^3^/µL), white blood cells 8.5 × 10^9^/L (2.9–28.2 × 10^9^/L), and D-dimer 8.8 µg/L (IQR 7.0–11.5 µg/L). One patient (5.6%) received a craniectomy and one an extraventricular drainage placement (5.6%). Thirteen (72.2%) patients were ventilated and eight (44.4%) patients received extracorporeal membrane oxygenation (ECMO) treatment. Sepsis and multiorgan failure (8 (44.4%)) were found in all death patients (mRS = 6) (8 (44.4%)), according to the autopsy diagnosis. In only two cases (11.1%), patients presented with neurological symptoms prior to COVID-19 associated respiratory symptoms. The majority of patients presented with COVID-19 associated respiratory symptoms prior to neurological symptoms (88.9%). The symptoms of COVID-19 were severe in most cases (83.3%). Three patients were excluded due to traumatic intracranial hemorrhage, and two patients were excluded due to hemorrhagic transformation following ischemic stroke.

### 3.2. Imaging and Clinical Findings 

**Figure 1:**
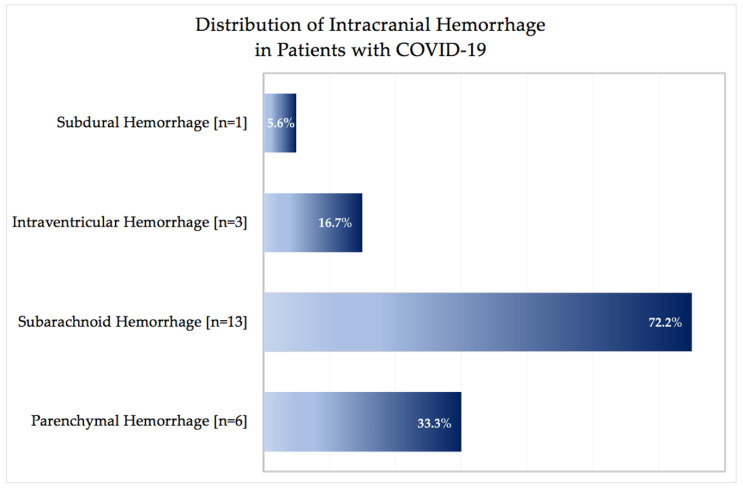
Distribution of intracranial hemorrhage in patients with Coronavirus Disease 2019. Legend: Distribution of intracranial hemorrhage events in patients with Coronavirus Disease 2019 (COVID-19). The distribution of the intracerebral hemorrhage type is presented in relation to all patients (*n* = 18).

Eighteen patients had evidence of intracranial hemorrhage on brain NCCT. Of these, two patients (11.1%) presented with intracranial hemorrhage and corresponding neurological symptoms at admission. Sixteen patients were diagnosed with intracranial hemorrhage (88.9%) during the course of hospitalization (Table 1). Figure 1 shows the distribution of intracranial hemorrhage types. Neuroimaging characteristics are displayed in Table 2. Six patients presented with parenchymal hemorrhage (33.3%) and 11 with SAH (61.1%). Out of the 11 patients with SAH, secondary SAH was seen in one patient with subdural hemorrhage that was accompanied by SAH (5.6%) and in one patient with parenchymal hemorrhage. Three patients had intraventricular hemorrhage (IVH) (16.7%) of which one was caused by secondary intraventricular extension in parenchymal hemorrhage. Isolated IVH did not extend to the surrounding parenchymal hemorrhage or infratentorial ventricular system (0.0%). No epidural hemorrhage was detected (0.0%).

**Figure 2:**
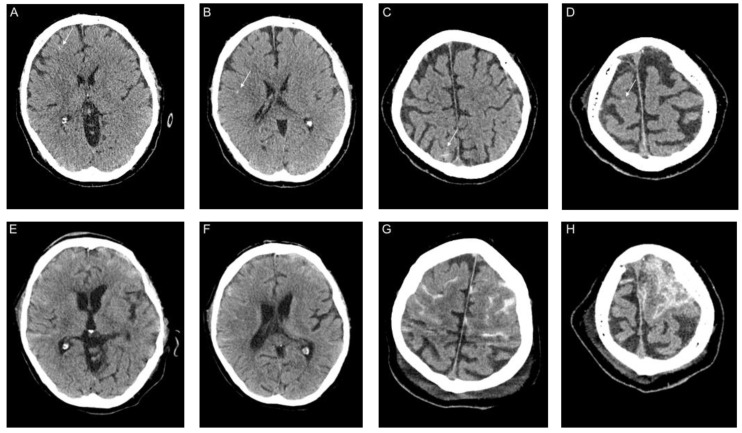
Representative computed tomography (CT) images of a patient with Coronavirus Disease 2019 with an acute subarachnoid hemorrhage (SAH). Legend: (**A**–**D**): Brain CT image two days after hospitalization. (**E**–**H**) Brain CT image seven days after hospitalization. White arrow indicates acute SAH.

**Figure 3:**
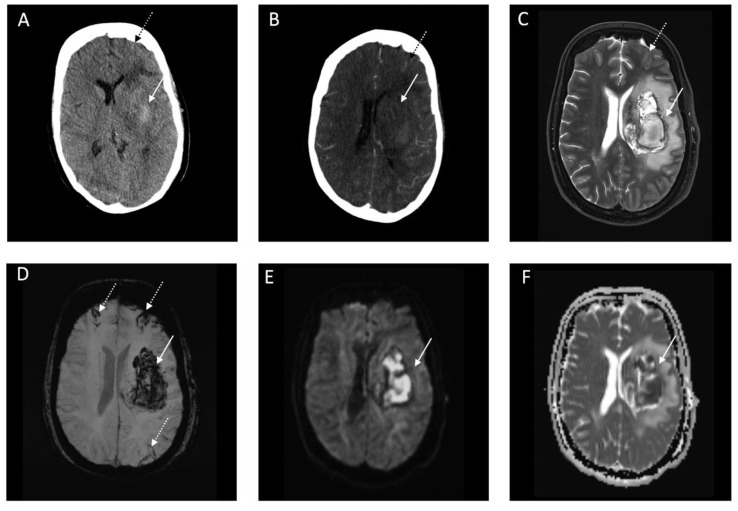
Representative computed tomography (CT) and Magnetic Resonance Imaging (MRI) images of a patient with Coronavirus Disease 2019 with a deep parenchymal hemorrhage and subarachnoid hemorrhage (SAH). Legend: (**A**): Brain CT image with corresponding computed tomography (CTA) image indicative of no underlying vascular pathology (**B**) at admission. (**C**–**F**): Brain MRI image one day after hospitalization with T2w (**C**), susceptibility weighted imaging (SWI) (**D**), diffusion weighted imaging (DWI) (**E**), and corresponding apparent diffusion coefficient (ADC) imaging map (**F**). White arrow indicates acute intracerebral hemorrhage. White-black dotted arrow indicates acute SAH.

**Figure 4:**
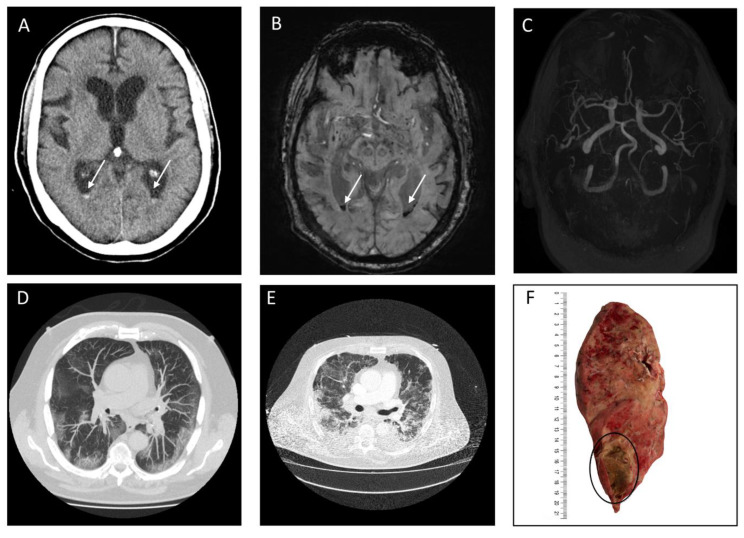
Representative computed tomography (CT) and Magnetic Resonance Imaging (MRI) images of a patient with Coronavirus Disease 2019 with an intraventricular hemorrhage (IVH). Legend: (**A**,**D**,**E**): Brain CT image (**A**) with corresponding chest CT image 30 days after hospitalization (**D**). (**B**,**C**,**E**): Brain MRI image with susceptibility weighted imaging (SWI) (**B**) and Time of flight angiography (TOF) (**C**) with corresponding chest CT image (**E**) 32 days after hospitalization. (**F**): Longitudinal section of the right lung indicative of purulent pneumonia with abscess formation in the lower lobe. White arrow indicates acute intraventricular hemorrhage and black circle indicates abscess formation.

**Figure 5:**
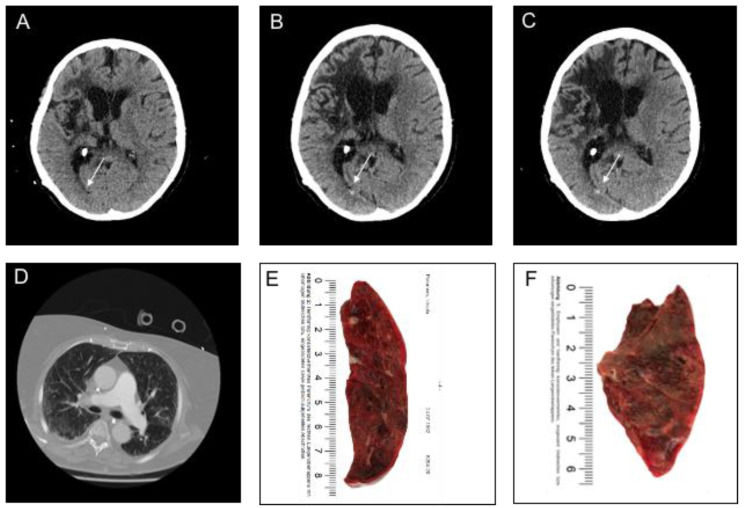
Representative computed tomography (CT) images of a patient with Coronavirus Disease 2019 with an intraventricular hemorrhage (IVH). Legend: (**A**,**D**): Brain CT image (**A**) with corresponding chest CT image at admission (**D**). (**B**,**C**): Brain CT image eight days (**B**) and ten days after hospitalization (**C**). (**F**): Longitudinal section of the right lung indicative of purulent, hemorrhagic pneumonia in the upper lobe. (**E**,**F**): Longitudinal section of the left lung indicative of purulent, hemorrhagic pneumonia and emphysematous structural alterations in the upper lobe. White arrow indicates acute intraventricular hemorrhage.

Clinical presentation of the 18 patients varied. One patient arrived at the emergency department with typical COVID-19 symptoms and GCS 13 (regular lung symptoms). The patient was neither ventilated nor under anticoagulation treatment at the time of admission imaging. Brain CT scan showed circumscribed cortical SAH across both hemispherical fissures with severe progress in the following days of hospitalization (Figure 2). One patient arrived at the emergency department owing to sudden onset of hemiplegia but without any typical symptoms (fever, cough, anorexia, and diarrhea) of COVID-19 (mild symptoms) (Figure 3). Emergent lung CT demonstrated typical COVID-19 lesion and the patient was also diagnosed as having COVID-19 by a positive SARS-CoV-2 PCR detection in the later stage. In the following days, the patient developed typical COVID-19 symptoms such as cough, throat pain with a ground-glass opacity appearance on lung CT. Two patients received additional MRI imaging following CT imaging (Figure 3 and Figure 4). One of the patients with MRI imaging presented with not only intraventricular hemorrhage but also disseminated microbleeds on susceptibility weighted imaging, which were linked to severe acute respiratory distress syndrome (severe symptoms) (Figure 4). No vascular pathologies were found in Time of flight angiography. An illustrative example of a second patient with IVH is presented in Figure 5. In addition, CTA was performed in 8 of the 18 cases without vascular pathologies, including vasculitis imaging features.

## 4. Discussion

This European multicenter study reports a total of 18 cases of acute intracranial hemorrhage in patients with confirmed COVID-19. Recent reports suggest the association of COVID-19 and intracranial hemorrhage, but the characteristics and mechanisms underlying the effect of SARS-CoV-2 infection remain poorly understood [3,7,12,13]. Although a direct association between the occurrence of acute intracranial hemorrhage and COVID-19 cannot be clearly demonstrated, it is likely to occur in patients with severe COVID-19 and a late temporal course. Evidence of intracranial hemorrhage in patients with COVID-19 was found within a median of 11 days of hospitalization with respiratory symptoms prior to neurological ones. A combination of acute intracranial hemorrhage and COVID-19 led to death in eight patients (44.4%). Isolated cortical SAH was the most frequent hemorrhage type followed by parenchymal hemorrhage (Figure 1). Isolated cortical SAH was localized in most cases along the convexity of the brain which is a relatively rare phenomenon [14]. Vascular disorders are the main underlying cause, including cortical vein thrombosis, vascular malformations, reversible cerebral vasoconstriction syndrome (RVCS), vasculitides, infectious aneurysms, reversible encephalopathy syndrome (PRES), and cerebral amyloid angiopathy [14]. PRES with secondary hemorrhage has recently been described in a series of severe COVID-19 infections with circumscribed parenchymal hemorrhage along the convexities [15,16]. Etiology of PRES can be diverse but is highly associated with sepsis, also found in 8 out of 18 patients in our case series (44.4%). Nevertheless, a separate consideration of RCVS is warranted as isolated SAH may be the presentation symptom in at least a quarter of these cases [17,18]. SAH in RVCS is mostly accompanied by multiple intracranial arterial focal stenoses [14,18]. Additional CTA in 8 out of the 18 patients showed no neurovascular pathologies. However, parenchymal ischemic or hemorrhagic lesions can also be seen on neuroimaging in up to 25% [14,18]. Both PRES and RCVS play a key role in endothelial dysfunction and the mechanisms of the observed high number of cortical SAH along the convexity might therefore be similar. A recent publication speaks in favor of a potential endothelial dysfunction as it reports a small case series of three COVID-19 patients with multiorgan failure and evidence of direct SARS-CoV-2 infection of the endothelial cells with diffuse endothelial inflammation [6]. In spite of the relatively small number of patients who underwent autopsy in our case series, the histopathological pattern was similar presenting with multiorgan failure (Figure 3 and Figure 4). In the brain, vascular endothelial cells primarily form the blood–brain–barrier (BBB) and maintain the interface between central nervous system and circulating blood [19]. Thereby, systemic vascular endotheliitis in COVID-19 may promote severe vessel weakening, blood vessel ruptures, and thus form a procoagulative state with subsequent intracerebral hemorrhage [6,20]. In favor of this explanation, COVID-19-associated coagulopathy (CAC) has recently been introduced, correlating with the disease severity and elevations in D-dimer levels (Table 1) [21].

Evidence of a systemic vascular inflammation is also increasing. In pediatric COVID-19 infections, the Kawasaki syndrome has been described in 15 cases of children with COVID-19 across the USA and Europe [22,23]. Viral-induced vasculitis is a well-known phenomenon in cases of Hepatitis B and C, and Varicella viruses [24]. The association is much less clear in other viruses such as Human immunodeficiency viruses or other Herpes family viruses [24]. ICH and SAH are not a rare presenting manifestation observed in primary central nervous vasculitis (CNS) or CNS secondary to other vasculitides [24,25,26,27]. In these patients, the histologic appearance of the brain biopsy confirmed an acute necrotizing pattern of vasculitis and may indicate related pathologic mechanisms to SARS-CoV-2 [24,25,28]. The major pathologic state of necrotizing vasculitis is typically associated with parenchymal hemorrhage. For instance, the referred series by Salvarani et al. found that parenchymal hemorrhage in patients with PCNS vasculitis was more common than SAH [25,29]. In our study, neurovascular imaging was performed in nearly half of the patients without evidence of a vascular pathology. Although the possibility of intracranial hemorrhage due to underlying vasculitis is valid even without evidence of vascular pathologies [30,31], we think other explanations should be entertained: The SARS-CoV-2 uses Angiotensin-converting enzyme 2 (ACE2) receptors expressed by pneumocytes in the epithelial alveolar lining to infect the host, thereby causing lung injury, but studies demonstrate also a wide expression of the ACE2 receptor on endothelial cells in the brain [6,32]. Expression of ACE2 receptors on endothelial cells could trigger a cytokine storm which recruits macrophages and causes inflammatory reactions, similar to those of vasculitis. Secondary SAH along the convexity as presented in one patient with lobar hemorrhage may also be found in some cases of cerebral amyloid angiopathy (CAA). According to the Boston Criteria, Cerebral Amyloid Angiopathy (CAA) was not considered as probable in our study as there was no appropriate clinical history of cognitive impairment and acute stroke symptoms accompanied by imaging findings of multiple cortical–subcortical hematomas, which may be of varying ages and sizes [33]. Moreover, ICH and SAH may be linked to arterial hypertension induced by binding of SARS-CoV-2 to ACE2 receptors and thrombocytopenia [34,35]. Arterial hypertension was found in more than 50% of the patients in our case series. Conclusively, an inflammatory phenomenon could also contribute, but could not be directly linked, to SARS-CoV-2. 

Despite major technological and medical improvements, ECMO and ventilation remain associated with incidences of intracranial hemorrhage [36,37]. In our study cohort, seven (41.2%) patients were under ECMO treatment. However, our case series shows very clearly that intracranial hemorrhage occurred prior to ECMO and ventilation therapy and hence may support the theory of a systemic vascular inflammation (Figure 2). Moreover, the severity of intracranial hemorrhage might be associated with the severity of COVID-19 infection, as one patient was admitted initially with circumscribed cortical and bihemispherical SAH with typical symptoms of non-severe COVID-19, but without anticoagulant or ventilatory treatment, and a GCS of 15. During the time of hospitalization, both cortical SAH and respiratory disease aggravated (Figure 2).

To our knowledge, this is the largest case series of patients with COVID-19 and accompanied intracerebral hemorrhage. Major strengths of this study include the heterogeneous sample of patients recruited from a range of hospitals in key European countries with geospatial hotspots of COVID-19, who were assessed according to a standardized protocol and objective measures [38]. In China, guidelines have been proposed to encourage neurologists to consider both ischemic and hemorrhagic strokes as potential complications of COVID-19 [39]. This study highlights the need for additional consensus guidelines for early recognition and treatment of intracranial hemorrhage in patients with COVID-19 [39]. Intensive blood pressure control and coagulopathy reversal may translate to a better clinical outcome in these patients [40,41]. Further, raised intracranial pressure (ICP) is related to mortality in comatose patients with intracranial hemorrhage, and ICP monitoring may be useful in guiding therapy, especially the timing and selection of patients for surgery [42].

We recognize several limitations, in particular the inclusion of a relatively small sample size and its retrospective nature. Due to the limited number of patients included, our study does not allow to draw definitive conclusions about the association of specific bleeding types and COVID-19. In addition, CTA was not performed routinely to further elucidate the presence of imaging features of vasculitis. An expansion of sample size in a prospective study design would certainly contribute to further understanding of the hemorrhage cause and in improving the generalizability of our results. Secondly, because most patients were still hospitalized and information regarding clinical outcomes was unavailable at the time of analysis, it was difficult to assess the effect of these neurologic manifestations on their outcome, and continued observations of the natural history of disease are needed. Finally, CNS biopsies were not available at the point of data acquisition.

## 5. Conclusions

In this European multicenter cohort, we present the up-to-date largest case series of patients with intracranial hemorrhage diagnosed with COVID-19. Our results suggest that the presence of intracranial hemorrhage may occur in a late temporal course of severe COVID-19 and prior to ECMO treatment. Isolated acute cortical SAH along the convexity was the most frequent presentation. Further research is needed to shed light on the underlying mechanisms.

## Figures and Tables

**Table 1:** Baseline demographic, clinical, and characteristics of patients with coronavirus disease 2019 (COVID-19).

Baseline Characteristics	COVID-19 Patients with Acute Intracranial Hemorrhage (*n* = 18)
Clinical Parameters	
Age (years), median (IQR)	49.50 (39.25–62.75)
Female, *n* (%)	9 (50.0)
Hypertension, *n* (%)	10 (55.6)
Diabetes mellitus, *n* (%)	4 (22.2)
Comorbidities	
• cardiovascular, *n* (%)	6 (33.3)
• neurovascular, *n* (%)	1 (5.6)
• oncological, *n* (%)	4 (22.2)
GCS score, median (IQR)	9 (3–7)
Anticoagulation Treatment, *n* (%)	8 (44.4)
Antiplatelet Treatment, *n* (%)	1 (5.6)
Time difference between symptom onset to imaging, (days), median (IQR)	1.5 (0–3)
Time difference between admission to imaging, (days), median (IQR)	11 (9–29.4)
Laboratory Parameters	
Lactate dehydrogenase, median (U/L), median (IQR)	483.50 (278–738.5)
Creatinine (mg/dL), median (IQR)	2.06 (1.45–2.81)
C-reactive protein (mg/L), median (IQR)	239.8 (145–377.14)
Platelet (10^3^/µL), median (IQR)	218.0 (108.5–445.25)
White blood cells [×10^9^/L], median (IQR)	8.5 (2.9–28.2)
D-dimer (µg/L), median (IQR)	8.8 (7.0–11.5)
COVID-19 Symptoms	
Neurological symptoms first, *n* (%)	2 (11.1)
Respiratory symptoms first, *n* (%)	16 (88.9)
COVID-19 Classification	
Mild lung infection, *n* (%)	1 (5.6)
Regular lung infection, *n* (%)	1 (5.6)
Severe lung infection, *n* (%)	1 (5.6)
Critical lung infection, *n* (%)	15 (83.3)
Procedure Process and Results	
Craniectomy, *n* (%)	1 (5.6)
EVD, *n* (%)	1 (5.6)
ECMO, *n* (%)	8 (44.4)
Ventilation, *n* (%)	13 (72.2)
Sepsis, *n* (%)	8 (44.4)
mRS, *n* (%)	
• 0–3	0 (0)
• 4–6	18 (100)

Legend: Comparison of baseline demographic, clinical characteristics in patients with COVID-19 and an acute intracranial hemorrhage. ECMO indicates extracorporeal membrane oxygenation; EVD, extraventricular drainage; GCS, Galsgow Coma Scale.

**Table 2:** Neuroimaging characteristics of patients with COVID-19.

Parenchymal Hemorrhage (*n* = 6)	
Location	
• Lobar, *n* (%)	4 (66.7)
• Deep, *n* (%)	1 (16.7)
• Infratentorial, *n* (%)	1 (16.7)
Ventricular extension, *n* (%)	1 (16.7)
SAH extension, *n* (%)	1 (0)
Subarachnoid Hemorrhage (*n* = 11)	
Primary SAH, *n* (%)	9 (81.8)
• Aneurysmal, *n* (%)	-
• Cortical, *n* (%)	9 (81.8)
• Bilateral, *n* (%)	4 (36.4)
• Ventricular extension, *n* (%)	-
Secondary SAH, *n* (%)	2 (18.2)
Intraventricular Hemorrhage (*n* = 3)	
Isolated IVH	
• Supratentorial, *n* (%)	3 (100)
• Infratentorial, *n* (%)	-
• SAH extension, *n* (%)	-
Secondary IVH ^*1^, *n* (%)	1 (33.3)
Subdural Hemorrhage (*n* = 1)	
Convexity, *n* (%)	1 (100)
Bilateral, *n* (%)	-
SAH extension, *n* (%)	1 (100)

Legend: Comparison of neuroimaging characteristics in patients with COVID-19 and an acute intracranial hemorrhage. The distribution of imaging characteristics are presented in relation to their subsequent intracerebral hemorrhage type. SAH indicates subarachnoid hemorrhage. Please note: ^*1^ = due to expansion in parenchymal hemorrhage.

## References

[1] WHO Coronavirus Disease (COVID-19) Dashboard. https://covid19.who.int/.

[2] Fu L., Wang B., Yuan T., Chen X., Ao Y., Fitzpatrick T., Li P., Zhou Y., Lin Y.F., Duan Q. (2020). Clinical characteristics of coronavirus disease 2019 (COVID-19) in China: A systematic review and meta-analysis. J. Infect..

[3] Mao L., Jin H., Wang M., Hu Y., Chen S., He Q., Chang J., Hong C., Zhou Y., Wang D. (2020). Neurologic manifestations of hospitalized patients with coronavirus disease 2019 in Wuhan, China. JAMA Neurol..

[4] Pinna P., Grewal P., Hall J.P., Tavarez T., Dafer R.M., Garg R., Osteraas N.D., Pellack D.R., Asthana A., Fegan K. (2020). Neurological manifestations and COVID-19: Experiences from a tertiary care center at the Frontline. J. Neurol. Sci..

[5] Lippi G., Favaloro E.J. (2020). D-dimer is associated with severity of coronavirus disease 2019: A pooled analysis. Thromb. Haemost..

[6] Varga Z., Flammer A.J., Steiger P., Haberecker M., Andermatt R., Zinkernagel A.S., Mehra M.R., Schuepbach R.A., Ruschitzka F., Moch H. (2020). Endothelial cell infection and endotheliitis in COVID-19. Lancet.

[7] Mahammedi A., Saba L., Vagal A., Leali M., Rossi A., Gaskill M., Sengupta S., Zhang B., Carriero A., Bachir S. (2020). Imaging in neurological disease of hospitalized COVID-19 patients: An Italian multicenter retrospective observational study. Radiology.

[8] Kerleroux B., Fabacher T., Bricout N., Moïse M., Testud B., Vingadassalom S., Ifergan H., Janot K., Consoli A., Ben Hassen W. (2020). Mechanical thrombectomy for acute ischemic stroke amid the COVID-19 outbreak. Stroke.

[9] Merkler A.E., Parikh N.S., Mir S., Gupta A., Kamel H., Lin E., Lantos J., Schenck E.J., Goyal P., Bruce S.S. (2020). Risk of ischemic stroke in patients with COVID-19 versus patients with influenza. JAMA Neurol..

[10] Novel Coronavirus Pneumonia Diagnosis and Treatment Plan (Provisional 7th Edition). https://www.chinalawtranslate.com/coronavirus-treatment-plan-7/.

[11] Broderick J.P., Adeoye O., Elm J. (2017). Evolution of the modified rankin scale and its use in future stroke trials. Stroke.

[12] Zulfiqar A.-A., Lorenzo-Villalba N., Hassler P., Andrès E. (2020). Immune thrombocytopenic purpura in a patient with COVID-19. N. Engl. J. Med..

[13] Akute Zerebrovaskuläre Ereignisse Bei COVID-19. https://www.dgn.org/rubrik-themen/3961-akute-zerebrovaskulaere-ereignisse-bei-covid-20.

[14] Cuvinciuc V., Viguier A., Calviere L., Raposo N., Larrue V., Cognard C., Bonneville F. (2010). Isolated acute nontraumatic cortical subarachnoid hemorrhage. Am. J. Neuroradiol..

[15] Princiotta Cariddi L., Tabaee Damavandi P., Carimati F., Banfi P., Clemenzi A., Marelli M., Giorgianni A., Vinacci G., Mauri M., Versino M. (2020). Reversible encephalopathy syndrome (PRES) in a COVID-19 patient. J. Neurol..

[16] Franceschi A.M., Ahmed O., Giliberto L., Castillo M. (2020). Hemorrhagic posterior reversible encephalopathy syndrome as a manifestation of COVID-19 infection. Am. J. Neuroradiol..

[17] Forman R., Harris J., Lee V., Garg R., John S., Conners J. (2017). Reversible cerebral vasoconstriction syndrome presenting as convexity subarachnoid hemorrhage (P2.286). Neurology.

[18] Barboza M.A., Maud A., Rodriguez G.J. (2014). Reversible Cerebral Vasoconstriction Syndrome and Nonaneurysmal Subarachnoid Hemorrhage. J. Vasc. Int. Neurol..

[19] Peeyush Kumar T., McBride D.W., Dash P.K., Matsumura K., Rubi A., Blackburn S.L. (2019). Endothelial cell dysfunction and injury in subarachnoid hemorrhage. Mol. Neurobiol..

[20] Bonetti P.O., Lerman L.O., Lerman A. (2003). Endothelial dysfunction: A marker of atherosclerotic risk. Arterioscler. Thromb. Vasc. Biol..

[21] Fogarty H., Townsend L., Cheallaigh C.N., Bergin C., Martin-Loeches I., Browne P., Bacon C.L., Gaule R., Gillett A., Byrne M. (2020). COVID-19 coagulopathy in caucasian patients. Br. J. Haematol..

[22] Jones V.G., Mills M., Suarez D., Hogan C.A., Yeh D., Segal J.B., Nguyen E.L., Barsh G.R., Maskatia S., Mathew R. (2020). COVID-19 and kawasaki disease: Novel virus and novel case. Hosp. Pediatrics.

[23] Verdoni L., Mazza A., Gervasoni A., Martelli L., Ruggeri M., Ciuffreda M., Bonanomi E., D’Antiga L. (2020). An outbreak of severe Kawasaki-like disease at the Italian epicentre of the SARS-CoV-2 epidemic: An observational cohort study. Lancet.

[24] Sharlala H., Adebajo A. (2008). Virus-induced vasculitis. Curr. Rheumatol. Rep..

[25] Salvarani C., Brown R.D., Calamia K.T., Christianson T.J.H., Huston J., Meschia J.F., Giannini C., Miller D.V., Hunder G.G. (2011). Primary central nervous system vasculitis presenting with intracranial hemorrhage. Arthritis Rheum..

[26] Boulouis G., De Boysson H., Zuber M., Guillevin L., Meary E., Costalat V., Pagnoux C., Naggara O. (2017). Primary angiitis of the central nervous system: Magnetic resonance imaging spectrum of parenchymal, meningeal, and vascular lesions at baseline. Stroke.

[27] Younger D.S., Coyle P.K. (2019). Central nervous system vasculitis due to infection. Neurol. Clin..

[28] Jain R., Deveikis J., Hickenbottom S., Mukherji S.K. (2003). Varicella-zoster vasculitis presenting with intracranial hemorrhage. Am. J. Neuroradiol..

[29] Salvarani C., Brown R.D., Hunder G.G. (2012). Adult primary central nervous system vasculitis. Curr. Opin. Rheumatol..

[30] Greenan T.J., Grossman R.I., Goldberg H.I. (1992). Cerebral vasculitis: MR imaging and angiographic correlation. Radiology.

[31] Marsh E.B., Zeiler S.R., Levy M., Llinas R.H., Urrutia V.C. (2012). Diagnosing CNS vasculitis. Neurologist.

[32] Hamming I., Timens W., Bulthuis M.L.C., Lely A.T., Navis G.J., van Goor H. (2004). Tissue distribution of ACE2 protein, the functional receptor for SARS coronavirus. A first step in understanding SARS pathogenesis. J. Pathol..

[33] Sharma R., Dearaugo S., Infeld B., O’Sullivan R., Gerraty R.P. (2018). Cerebral amyloid angiopathy: Review of clinico-radiological features and mimics. J. Med. Imaging Radiat. Oncol..

[34] Guan W., Ni Z., Hu Y., Liang W., Ou C., He J., Liu L., Shan H., Lei C., Hui D.S.C. (2020). Clinical characteristics of coronavirus disease 2019 in China. N. Engl. J. Med..

[35] Oudit G.Y., Kassiri Z., Jiang C., Liu P.P., Poutanen S.M., Penninger J.M., Butany J. (2009). SARS-coronavirus modulation of myocardial ACE2 expression and inflammation in patients with SARS. Eur. J. Clin. Investig..

[36] Cavayas Y.A., del Sorbo L., Fan E. (2018). Intracranial hemorrhage in adults on ECMO. Perfusion.

[37] Fanou E.M., Coutinho J.M., Shannon P., Kiehl T.R., Levi M.M., Wilcox M.E., Aviv R.I., Mandell D.M. (2017). Critical illness-associated cerebral microbleeds. Stroke.

[38] Mavragani A. (2020). Tracking COVID-19 in Europe: Infodemiology approach. JMIR Public Health Surveill..

[39] Jin H., Hong C., Chen S., Zhou Y., Wang Y., Mao L., Li Y., He Q., Li M., Su Y. (2020). Consensus for prevention and management of coronavirus disease 2019 (COVID-19) for neurologists. Stroke Vasc. Neurol..

[40] Hemphill J.C., Greenberg S.M., Anderson C.S., Becker K., Bendok B.R., Cushman M., Fung G.L., Goldstein J.N., MacDonald R.L., Mitchell P.H. (2015). Guidelines for the Management of Spontaneous Intracerebral Hemorrhage: A Guideline for Healthcare Professionals from the American Heart Association. Stroke.

[41] Sweidan A.J., Singh N.K., Conovaloff J.L., Bower M., Groysman L.I., Shafie M., Yu W. (2020). Coagulopathy reversal in intracerebral haemorrhage. Stroke Vasc. Neurol..

[42] Ropper A.H., King R.B. (1984). Intracranial pressure monitoring in comatose patients with cerebral hemorrhage. Arch. Neurol..

